# Incidence of Malaria in the Interior Division of Sabah, Malaysian Borneo, Based on Nested PCR

**DOI:** 10.1155/2011/104284

**Published:** 2011-10-13

**Authors:** Wan Fen Joveen-Neoh, Ka Lung Chong, Clemente Michael Vui Ling Wong, Tiek Ying Lau

**Affiliations:** Biotechnology Research Institute, Universiti Malaysia Sabah, Jalan UMS, 88400 Kota Kinabalu, Sabah, Malaysia

## Abstract

*Introduction*. Malaria is currently one of the most prevalent parasite-transmitted diseases caused by parasites of the genus *Plasmodium*. Misidentification of human malaria parasites especially *P. knowlesi *based on microscopic examination is very common. The objectives of this paper were to accurately identify the incidence of human malaria parasites in the interior division of Sabah, Malaysian Borneo, based on small subunit ribosomal RNA (*ssrRNA*) and to determine the misidentification rate in human malaria parasites. *Methods*. Nested PCR was used to detect the presence of human malaria parasites. A total of 243 blood spot samples from patients who had requested for blood film for malaria parasite (BFMP) analyses were used in this study. *Results*. Nested PCR findings showed that there was no *P. malariae *infection while the highest prevalent malaria parasite was *P. knowlesi*, followed by *P. vivax*, *P. falciparum*, and mixed infection. Only 69.5% of the 243 samples giving consistent nested PCR and microscopic results. *Conclusion*. The preliminary findings from molecular detection of malaria showed that *P. knowlesi *was the most prevalent *Plasmodium *species in the interior division of Sabah. The findings from this paper may provide a clearer picture on the actual transmission of different *Plasmodium *species in this region.

## 1. Introduction

Malaria is a tropical disease caused by parasites of the genus *Plasmodium*. Around 250 million of malaria cases and one million deaths caused by malaria are reported around the world, especially in underdeveloped and remote regions [1]. In most South East Asia countries, malaria remains a serious threat to public health [2]. Furthermore, this disease is the most common vector-borne parasitic disease in remote areas of Malaysia [3]. 

Four *Plasmodium* parasites, namely, *Plasmodium falciparum*, *P. vivax*, *P. malaria,* and *P. ovale*, are well recognized to cause malaria worldwide. Recently, *P. knowlesi* has been acknowledged as the “fifth human malaria species” following its discovery in humans in Malaysian Borneo [4]. Since then, naturally acquired *P. knowlesi* infection was found to be widespread in Malaysian Borneo and Pahang in West Malaysia [5]. 

Routine microscopic examination has been considered as the “gold standard” for the diagnosis of *Plasmodium* parasite infection due to its simple, rapid, and cost-effective features [6]. Nevertheless, this method is prone to misdiagnosis in the cases of mixed infections and low-level parasitemia [7]. The aims of this study were to accurately identify the malaria parasites in humans and to determine the numbers of misidentification in *Plasmodium* parasites in the interior division of Sabah using molecular techniques. The presence of *Plasmodium* species parasite in patient samples prediagnosed using microscopic methods were subjected to detection using the nested PCR. Amplification was carried out using genus- and species-specific primers targeting the small subunit ribosomal RNA gene (*ssrRNA*) of the parasites. This PCR-based assay had been used successfully for detection of low-level parasitemia and accurate diagnosis of malaria parasite species [8].

## 2. Materials and Methods

### 2.1. Study Sites

**Figure 1:**
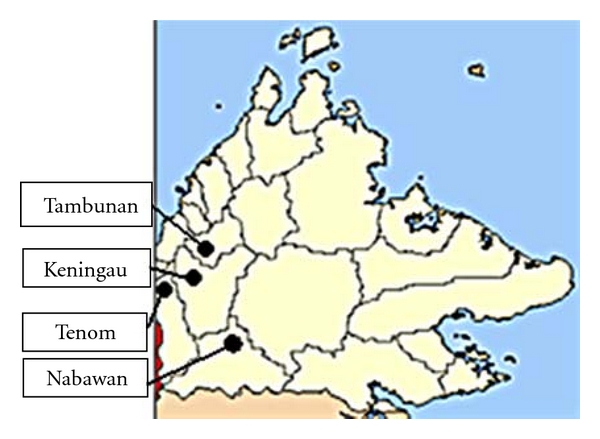
Map of the Sabah state indicating the location of the study sites in this study.

The state of Sabah covers an area of 76,114.92 km^2^ and is situated on the northern part of Malaysian Borneo. Samples were collected from Keningau, Tambunan, Tenom, and Nabawan in the interior division of Sabah which is hilly, surrounded by forest fringe, and the Crocker Range (Figure 1). Study sites are covered with primary and secondary jungles, habitats that are suitable for natural hosts for *P. knowlesi* such as the long and pig-tailed macaques and the vectors (*Anopheles leucosphyrus* group of mosquitoes) [9]. 

### 2.2. Sample Collection

Ethical clearance were obtained from the Ethical Committee of Ministry of Health Malaysia and from the Ethical Committee of Universiti Malaysia Sabah (UMS) to conduct this study and for blood sample collection. Besides, patients' consent were obtained prior to blood sample collection. Blood samples were collected from February to November in year 2010 from patients at the Hospitals of Keningau, Tenom, Tambunan, and Klinik Kesihatan Nabawan. The target groups were those whom were suspected to be infected with malaria and had requested blood film for malaria parasites (BFMP). Sample collections were carried out by the qualified medical laboratory staffs of the respective hospitals. Three spots with total volume of 25 *μ*L of blood each were spotted onto chromatography papers (Whatman 3MM) and dried.

Blood samples taken from healthy individuals with no history of malaria infection were used as negative control in this study. 

Infected blood samples and the parasites causing the infections were recorded based on the routine diagnosis reports prepared by medical laboratory staffs of the respective hospitals. The presence of *Plasmodium* species in the Giemsa-stained thin-blood film was determined microscopically by the qualified medical laboratory staffs.

### 2.3. Extraction of Genomic DNA

The extraction of genomic DNA from dried blood spots on the filter paper were performed using the InstaGene Matrix (Bio-Rad Laboratories, Inc., CA, USA) method as described [10]. One negative control was included in each batch of extraction to ensure that no contamination occurred during DNA extraction. The extracted DNA was kept in −20°C until further use.

### 2.4. Detection of Malaria Parasites by Nested PCR

Identification of *Plasmodium* species was conducted using *Plasmodium* genus- and species-specific PCR based on small subunit ribosomal RNA (*ssrRNA*) gene as previously described [11]. For each PCR reaction, positive control for each *Plasmodium* species and negative control were included. 

Nest 1 *Plasmodium* genus-specific PCR amplification was performed in 50 *μ*L reaction mixtures containing 1 X Promega GoTaq Flexi Buffer, 3 mM of MgCl_2_, 0.2 mM of each deoxynucleoside triphosphate (dNTP), 0.25 *μ*M of each primer (rPLU1 and rPLU5), 1.25 unit *Taq* DNA Polymerase (Promega Corporation, USA), 15 *μ*L of genomic DNA template, and the final volume was adjusted to 50 *μ*L with molecular grade water. Cycling condition of nest 1 PCR amplification of *Plasmodium ssrRNA* was as follows; initial denaturation at 94°C for 4 min, 30 cycles of denaturation at 94°C for 30 sec, annealing at 55°C for 1 min, and extension at 65°C for 1 min 30 sec, followed by final extension at 65°C for 5 min.

Two *μ*L of the nest 1 amplification product was used as the template DNA in the nest 2 PCR amplification. Concentration of the constituents and nest 2 primers were similar with nest 1 except that 2 mM of MgCl_2_ and 0.5 units of *Taq* DNA polymerase were used. Conditions of nest 2 amplification were similar to those of nest 1 except for the annealing temperature which was 58°C for genus-specific primers (rPLU3 and rPLU4) and 62°C for species-specific primers (rFAL1/rFAL2, rVIV1/rVIV2, rMAL1/rMAL2, pmk8/pmk9, and rOVA1/rOVA4) [11]. 

The nest 2 PCR amplification products were analyzed by gel electrophoresis in a 2.7% agarose gel and visualized by staining with ethidium bromide (4 *μ*g/mL) and ultraviolet transillumination.

## 3. Results

### 3.1. Study Population

A total of 243 blood samples were collected in a period of six months. Samples were contributed mainly by male patients (74.5%) and the highest number of samples was collected from patients in the age group 11–20 years old comprising 62 (25.5%) samples. Among the four study sites, Tambunan has the highest cases reported (87 cases) whereas only 45 cases were reported in Tenom.

### 3.2. Prevalence of *Plasmodium* Species Detected by Microscopic Examination

Of the total 243 samples, 83 (34.2%) samples were positive for malarial parasite and the remaining samples were negative for malaria parasite based on microscopic finding. These were constituted of 39 *Plasmodium malariae *samples (16.1%), 25 (10.3%) *P. vivax* samples, 17 (7%) *P. falciparum* samples, and three (1.2%) samples with mixed infection (*P. falciparum* and *P. malariae*). There was no *P. ovale* and *P. knowlesi* malaria reported from the microscopic examination. 

**Figure 2:**
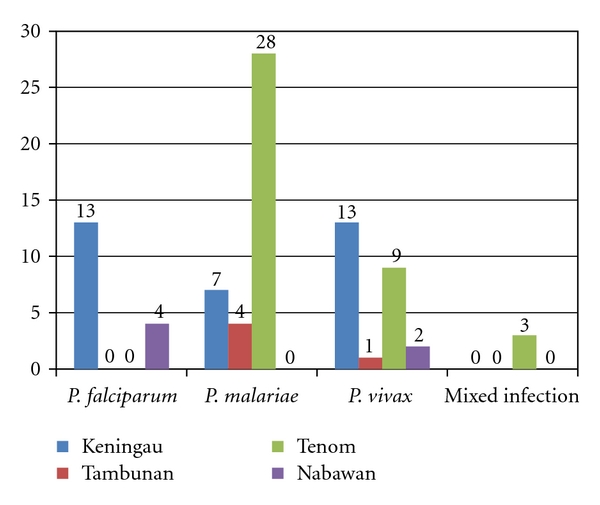
Distribution of *Plasmodium* species according to study sites based on microscopic findings. The numbers in the chart indicated the number of cases of *Plasmodium* species in each study site.

Based on microscopic findings, Keningau had the highest incidence of *P. falciparum* (13 cases), followed by four cases from Nabawan. The incidence of *P. malariae* was highest in Tenom with 28 samples followed by seven samples from Keningau and four cases reported from Tambunan. Besides, the incidence of *P. vivax* was highest in Keningau, where 13 *P. vivax* samples were detected microscopically. This was followed by nine *P. vivax* cases from Tenom, two *P. vivax* cases from Nabawan, and one *P. vivax* infection from Tambunan. In addition, three mixed infection of *P. falciparum* and *P. malariae* cases were also reported from Tenom (Figure 2). 

### 3.3. Detection of Malaria Parasites by Nested PCR

**Figure 3:**
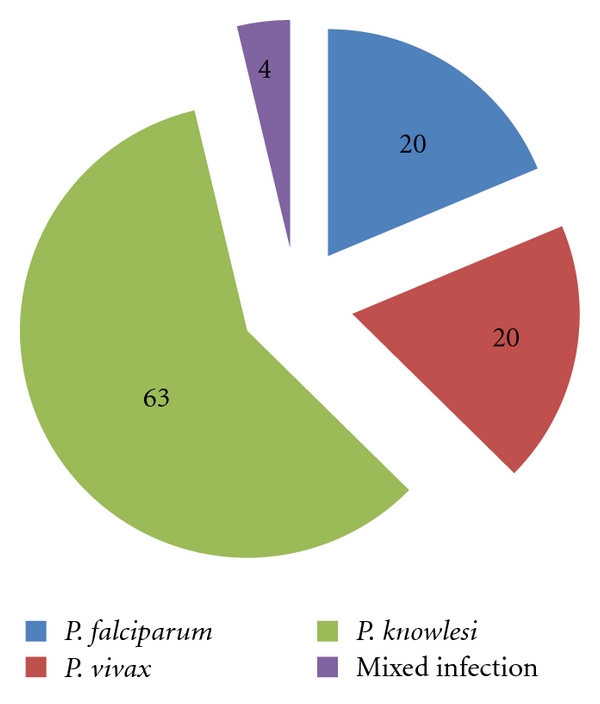
Authentication of *Plasmodium* species in the interior division of Sabah. The numbers in the pie chart denoted number of positive cases.

A total of 107 samples were positive for *Plasmodium* genus by genus-specific nested PCR. Sixty-three (63) of the samples were infected with *P. knowlesi* while 20 of the blood samples were infected with *P. vivax* and *P. falciparum* each. Besides, four samples had mixed infection (two *P. falciparum*/*P. vivax*, one *P. falciparum*/*P. knowlesi,* and one *P. vivax*/*P. knowlesi*) were also detected in this study. No *P. malariae* or *P. ovale* was detected using species-specific nested PCR (Figure 3).

Higher prevalence of *Plasmodium* species were detected by nested PCR compared to microscopic examination. As shown in Table 1, a total of 23 negative samples from microscopic finding were found to be positive for *Plasmodium* species by nested PCR detection. Besides, one microscopically *P.vivax* positive sample found to be negative for *Plasmodium* species by nested PCR.

### 3.4. Distribution of Malaria Cases according to Geographical Location

**Figure 4:**
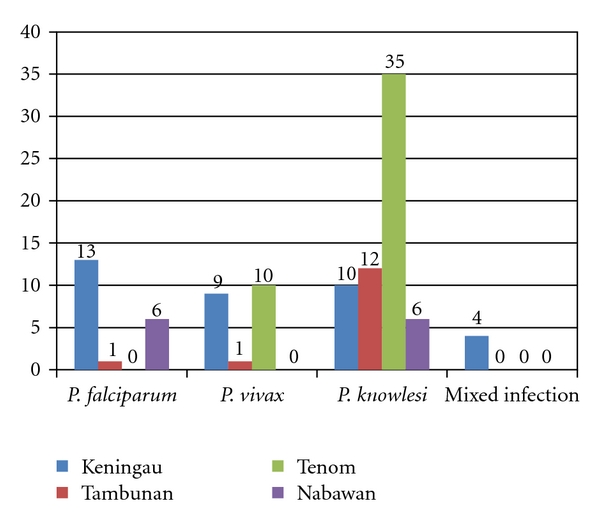
Distribution of human malaria parasites in the interior division of Sabah based on nested PCR. Numbers in the chart denoted number of cases detected.

Our study showed that the incidence of different *Plasmodium* species was not evenly distributed across the geographical regions in the interior division Sabah. Nested PCR showed that the highest *P. falciparum* infections were reported from Keningau (13 cases), followed by six cases in Nabawan, and one case from Tambunan. The highest incidence of *P. vivax* was reported in Tenom (ten cases), followed by nine cases in Keningau, and one infection from Tambunan. Nested PCR had successfully detected one *P. vivax* infection from a microscopically negative sample from Tenom while nine samples had consistent result with microscopic finding. In addition, one *P. vivax* was detected from a microscopically negative sample from Keningau on top of the eight *P. vivax* samples which showed consistency in both the microscopic and PCR findings. There was only one *P. vivax* case reported from Tambunan based on both microscopic examination and PCR detection. Besides, nested PCR findings showed that *P. knowlesi* infections were detected in all the four study sites. Tenom has the highest *P. knowlesi* (35 cases) infection, followed by 12 cases in Tambunan, ten cases in Keningau, and six cases in Nabawan (Figure 4). 

### 3.5. Comparison of *Plasmodium* Species Detection Based on Microscopic Examination and Nested PCR

There were more positive samples (107) for *Plasmodium* species detected by nested PCR as compared to microscopic examination (84). Twenty-three microscopically negative samples showing positivity for *Plasmodium* species were indicated by nested PCR. The species-specific nested PCR showed that five microscopic negative samples were detected as *P. falciparum*. Besides, two *P. vivax* samples were misidentified as negative by microscopic examination. In addition, there were 16 microscopic negative samples detected as single *P. knowlesi* infection by nested PCR. Furthermore, 40 *P. malariae* infections by microscopic examination were detected as *P. knowlesi* by nested PCR. Besides, three samples which reported as mixed infections of *P. falciparum* and *P. malariae* by microscopic examination were identified as single *P. knowlesi* by nested PCR. Nevertheless, nested PCR was also sensitive enough to detect mixed infection (*P. vivax*/*P. falciparum*, *P. vivax*/*P. knowlesi,* and *P. falciparum*/*P. knowlesi*) in four samples which were reported as single infection of *P. vivax* or *P. falciparum* microscopically. However, there was also one false *P. vivax* positive sample by microscopic examination showing negative for all human malaria parasite by nested PCR (Table 2).

## 4. Discussion

Four sample collection areas in this study; Keningau, Nabawan, Tambunan, and Tenom are located in the interior division of Sabah and is surrounded by Crocker Range and forests, suitable habitats for the malaria vectors and hosts. The primary rainforest also supports the population of nonhuman primate, which is the host for *Plasmodium* parasites. 

Nested PCR was used in detecting malaria parasites and identification of five human *Plasmodium* species in this study. The nested PCR was useful in detecting the *ssrRNA* gene sequence of human *Plasmodium* species due to the uniqueness of the sequences among *Plasmodium* species [12]. Amplification by nested PCR was efficient in such a way that the amount of product obtained does not alter the range of parasite DNA in the original sample [11, 13]. 

According to Ministry of Healthy Malaysia, *P. vivax* was the most prevalent human malaria parasite in Malaysia (Country Updates Malaysia, 2010). However, our study showed that the most prevalent *Plasmodium* species in the interior division was *P. knowlesi*, followed by *P. falciparum* and *P. vivax*. There was no *P. malariae* infection detected despite the presence of *P. malariae* positive samples (16%) by microscopic examination.

All infections diagnosed as *P. malariae* by microscopic examination were found to be *P. knowlesi* or nonmalaria *Plasmodium* species by nested PCR. *Plasmodium knowlesi *infection in humans is not new, but it has probably been misidentified as *P. malariae* or *P. falciparum* using the conventional microscopic methods. This has occurred for quite some time due to the similar morphological characteristics of *P. knowlesi* parasites with *P. falciparum* in early trophozoite stage and *P. malariae* in the later stages of erythrocytic cycle [14, 15]. Besides, recent findings also reported large number of *P. knowlesi* infection in Sarawak, Malaysian Borneo [16] and other regions in Southeast Asia such as Myanmar [17], Thailand [18], the Philippines [19], and Singapore [20]. Hence, the incidence of *P. knowlesi* in humans was not as rare as previously thought. Several reasons had been postulated regarding the sudden switch of host for *P. knowlesi* infection from long-tailed macaque to human. Human disturbance of the large tracts in the natural transmission sites for *P. knowlesi* and habitat destruction are one of the reasons [21]. Previously, human host niche which was already occupied and due to the lack of opportunity had prevented the entry of *P. knowlesi* into the human population [22, 23]. The geographical location of the selected study sites which are located in the forest fringe might explain the high incidence of *P. knowlesi* in this region. 

Throughout the study period, *P. knowlesi* infection was mainly detected in the samples collected from the four study regions. Prevalence of *P. knowlesi* infection in the interior division of Sabah was highest in the district of Tenom while its prevalence was lowest in Nabawan. Besides, among the four study sites, *P. falciparum* was the most prevalent *Plasmodium* species in Keningau. Previous studies had shown that *P. knowlesi* infection had been detected throughout Malaysian Borneo (Sarawak and Sabah) and a zoonosis masked parasite [14, 15]. Our study further proved that *P. knowlesi* was widely distributed in the interior division of Sabah. 

In comparing the detection of human malaria parasite, nested PCR was found to more superior than microscopy detection. By using nested PCR, more *Plasmodium* species were detected as compared to microscopic examination. PCR findings showed that 24 microscopically negative samples were detected as single infection of *P. falciparum* (5 cases), *P. vivax* (2 cases), and 17 samples of *P. knowlesi*. Circulating parasites and low level of parasitemia on the blood film might have caused the failure in detection of *Plasmodium* species. Besides, there was higher *P. knowlesi* infections misdiagnosed as negative for *Plasmodium* species by microscopic examination. Previous study had shown that multiple-infected erythrocytes were only commonly observed from the blood films with the parasitemia above 100,000 parasites/*μ*L blood [14, 15]. Similar and small size of the parasites' morphology as well as the difficulty in staining the parasites for observation and detection might also contribute to the misdiagnosis [7]. 

There were four mixed infections (*P. falciparum*/*P. knowlesi*, *P. falciparum/P. vivax *and *P. vivax/P. knowlesi*) detected by PCR from the singly infected *P. vivax,* and *P. falciparum* sample by microscopy. This might be due to the presence of higher number of parasites of one species relative to the other which cause the failure in detection of mixed infections by microscopic examination. *Plasmodium vivax* might be higher in number compared to *P. falciparum* which result in failure to detect *P. falciparum* by microscopic examination for the *P. vivax*/*P. falciparum* positive samples.

Nested PCR is a necessity in the detection of *P. knowlesi* [14, 15]. There were no *P. knowlesi* positive cases detected by microscopic examination in this study due to the difficulty to distinguish the morphology of *P. knowlesi* from *P. malariae*. Therefore, it was recommended that symptomatic malaria with hyperparasitemia and parasite similar to *P. malariae* microscopically to be diagnosed as *P. knowlesi* in Malaysia [2]. In summary, the misdiagnosis of *Plasmodium* species was found in 74 of 243 (30.5%) samples in this study. High rate of misidentification showed that the detection of *Plasmodium* species with microscopic examination was not sensitive and specific compared with nested PCR. This is crucial as the accurate diagnosis of *Plasmodium* species is important for proper treatment and disease management. 

This study highlighted the high incidence of *P. knowlesi* in the interior division of Sabah as detected by nested PCR which were misdiagnosed as *P. malariae* using conventional microscopic technique. PCR-based technique was found to be more sensitive and specific than microscopic examination for accurate diagnosis of malaria. A larger scale of epidemiological study focusing on to the forest-fringe areas can be carried out in the future to determine the actual transmission of malaria parasites especially *P. knowlesi* in Sabah. This finding is important for the effective control and treatment of malaria, especially the high incidence of *P. knowlesi*, a potential lethal *Plasmodium* parasite.

## Figures and Tables

**Table 1:** Detection of malaria parasite based on PCR and microscopic examination.

Method of detection	Positive	Negative	Total
Microscope	84	159	243
Nested PCR	107	136	243

**Table 2:** Comparison of *Plasmodium* species detection by nested PCR and microscopic examination.

*Plasmodium* species	Nested PCR	Microscopic examination
*P. falciparum*	20	17
*P. vivax*	20	25
*P. malariae*	0	39
*P. knowlesi*	63	0
*P. falciparum/P. malariae*	0	3
*P. falciparum/P. vivax*	2	0
*P. falciparum/P. knowlesi*	1	0
*P. knowlesi/P. vivax*	1	0
Negative	136	159

Total of positive samples	243	243

## References

[1] WHO (2009). World malaria report 2009.

[2] Vythilingam I, Chan ST, Shanmugratnam C, Tanrang H, Chooi KH (2005). The impact of development and malaria control activities on its vectors in the Kinabatangan area of Sabah, East Malaysia. *Acta Tropica*.

[3] Singh B, Cox-Singh J (2001). Parasites that cause problems in Malaysia: soil-transmitted helminths and malaria parasites. *Trends in Parasitology*.

[4] White NJ (2008). *Plasmodium knowlesi*: the fifth human malaria parasite. *Clinical Infectious Diseases*.

[5] Cox-Singh J, Davis TME, Lee KS (2008). *Plasmodium knowlesi* malaria in humans is widely distributed and potentially life threatening. *Clinical Infectious Diseases*.

[6] Singh B (1997). Molecular methods for diagnosis and epidemiological studies of parasitic infections. *International Journal for Parasitology*.

[7] Genc A, Eroglu F, Koltas IS (2010). Detection of *Plasmodium vivax* by nested pcr and real-time PCR. *Korean Journal of Parasitology*.

[8] Aslan G, Seyrek A, Kocagoz T, Ulukanligil M, Erguven S, Gunalp A (2007). The diagnosis of malaria and identification of *Plasmodium* species by polymerase chain reaction in Turkey. *Parasitology International*.

[9] Garnham PCC (1966). *Malaria Parasites and Other Haemosporidia*.

[10] Cox-Singh J, Mahayet S, Abdullah MS, Singh B (1997). Increased sensitivity of malaria detection by nested polymerase chain reaction using simple samplings and DNA extraction. *International Journal for Parasitology*.

[11] Snounou G, Viriyakosol S, Zhu XP (1993). High sensitivity of detection of human malaria parasites by the use of nested polymerase chain reaction. *Molecular and Biochemical Parasitology*.

[12] Snounou G, Singh B (2002). Nested PCR analysis of *Plasmodium* parasites. *Methods in Molecular Medicine*.

[13] Black J, Hommel M, Snounou G, Pinder M (1994). Mixed infetions with *Plasmodium falciparum* and *P. malariae* and fever in malaria. *The Lancet*.

[14] Lee KS, Cox-Singh J, Singh B (2009). Morphological features and differential counts of *Plasmodium knowlesi* parasites in naturally acquired human infections. *Malaria Journal*.

[15] Lee KS, Cox-Singh J, Brooke G, Matusop A, Singh B (2009). *Plasmodium knowlesi* from archival blood films: further evidence that human infections are widely distributed and not newly emergent in Malaysian Borneo. *International Journal for Parasitology*.

[16] Singh B, Sung LK, Matusop A (2004). A large focus of naturally acquired *Plasmodium knowlesi* infections in human beings. *The Lancet*.

[17] Zhu HM, Li J, Zheng H (2006). Human natural infection of *Plasmodium knowlesi*. *Chinese Journal of Parasitology &amp; Parasitic Diseases*.

[18] Jongwutiwes S, Putaporntip C, Iwasaki T, Sata T, Kanbara H (2004). Naturally acquired *Plasmodium knowlesi* malaria in human, Thailand. *Emerging Infectious Diseases*.

[19] Luchavez J, Espino F, Curameng P (2008). Human infections with *Plasmodium knowlesi*, the Philippines. *Emerging Infectious Diseases*.

[20] Ng OT, Eng EO, Cheng CL (2008). Naturally acquired human *Plasmodium knowlesi* infection, Singapore. *Emerging Infectious Diseases*.

[21] Macaulay V, Hill C, Achilli A (2005). Single, rapid coastal settlement of Asia revealed by analysis of complete mitochondrial genomes. *Science*.

[22] Conway DJ (2007). Molecular epidemiology of malaria. *Clinical Microbiology Reviews*.

[23] Douradinha B, Mota MM, Luty AJF, Sauerwein RW (2008). Cross-species immunity in malaria vaccine development: two, three, or even four for the price of one?. *Infection and Immunity*.

